# Self-Induced Traumatic Macroglossia: Case Report and Literature Review

**DOI:** 10.1155/2019/6040354

**Published:** 2019-05-12

**Authors:** Abdullah A. Alarfaj, Ali R. AlHayek, Rashid Alghanim, Nasser A. Al-Jazan

**Affiliations:** ^1^King Faisal University, Department of Otolaryngology, Hofuf, Saudi Arabia; ^2^Imam Abdulrahman Bin Faisal University, King Fahad Hospital of University, Khobar, Saudi Arabia

## Abstract

Traumatic macroglossia is an extremely rare condition characterized by a sudden edematous swelling of the tongue due to trauma. We report a rare case of traumatic macroglossia in a 37-year-old male with known trisomy 21 and epilepsy who presented to the emergency room with a huge protruded tongue due to aggressive behavior and a history of multiple tongue tractions, leading to sudden severe tongue swelling without any respiratory distress symptoms. The examination was unremarkable; fixable nasolaryngoscopy relieved bilateral vocal cord movement, and there was no laryngeal edema. The patient was managed immediately by endotracheal intubation to secure the airway, and corticosteroids were used to diminish and stop the tongue swelling. We describe the clinical management for such patients, highlighting the different causes of traumatic macroglossia. A few cases have been reported in the literature, but this is the first case to report self-induced traumatic macroglossia in a seizure-free patient managed successfully by endotracheal intubation, corticosteroids, a bite block, and warm wet dressing.

## 1. Introduction

Macroglossia is a rare condition defined as a protrusion of an enlarged tongue beyond the teeth or alveolar ridge in a natural resting position. Based on pathophysiology, it may develop due to tissue overgrowth or tissue infiltration. It has been classified by Myer and Vogel according to the extent and etiology of macroglossia. Based on the extent of involvement, Myer classifies macroglossia into generalized or localized [1]. Meanwhile, the Vogel classification divides macroglossia into true or relative based on etiology [2]. When the cause is a primary disorder of the tongue tissue, it is named true macroglossia, and when affected secondarily, such as by amyloidosis, it is termed relative macroglossia. However, there are many causes of macroglossia; it may be present since birth (congenital) or it could be acquired. Inherited or congenital disorders associated with macroglossia include various syndromes (e.g., Down syndrome and Beckwith-Wiedemann syndrome), hemangioma, myxedema, mucopolysaccharidosis, and neurofibromatosis [3]. Acquired causes may include metabolic or endocrine conditions, such as hypothyroidism, amyloidosis, and acromegaly; inflammatory/infectious diseases, such as pemphigus vulgaris, diphtheria, tuberculosis, and sarcoidosis; and trauma, as in our case. Moreover, neoplastic disorders may also cause macroglossia, such as lymphangioma or various malignancies (cancers). Although it may be asymptomatic, symptoms usually are more likely to be present and more severe with greater tongue enlargements. Signs and symptoms may consist of drooling, speech impairment, dysphagia, stridor, snoring, airway obstruction, abnormal growth of the jaw and teeth, ulceration, or tip necrosis.

Macroglossia is a clinical diagnosis. Therefore, a clinical history, examination, and basic imaging techniques will be sufficient to identify the etiology. Furthermore, nasopharyngeal endoscopy may be necessary to assess whether the upper airway is obstructed. However, at certain times, genetic and chromosomal studies may be needed to establish the diagnosis. To identify the dimensions, as well as the tumor margins, magnetic resonance imaging (MRI) is the best method of choice. In certain cases, a biopsy may be needed to identify the cause, such as in lingual thyroid and amyloidosis cases [4].

Treatment depends on the particular cause of macroglossia and its severity; it might range from speech therapy in a mild condition to surgical reduction (partial glossectomy) in a severe condition. Nevertheless, securing the airway is the first priority in the management of macroglossia in case of impulsive enlargement.

Traumatic macroglossia is a hematoma or edematous swelling of the tongue caused by an injury or trauma [5]. It has been reported in such cases as following palatal surgery using a Dingman mouth gag [6], trauma to the tongue in coagulopathic/anticoagulated patients, tongue biting in epileptic patients [7], oral cavity surgery, and prolonged orotracheal intubation, which causes venous/lymphatic obstruction [8]. Self-inflicted traumatic macroglossia is an extremely rare condition with few cases reported in the literature [7, 9, 10], but it is a life-threatening condition subsequent to sudden upper airway obstruction. Therefore, an emergent medical and surgical intervention is crucial in its management, and tracheostomy should be considered if endotracheal intubation fails. We report a case of self-induced traumatic macroglossia in a mentally handicapped patient who was treated with intravenous corticosteroids, and rapid endotracheal intubation was established in a controlled setting before an upper airway obstruction developed.

## 2. Case Report

**Figure 1:**
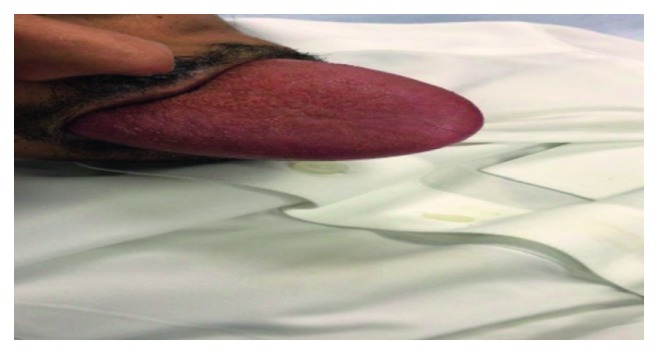
Clinical photograph of the patient in hospital following presentation with an anterior tongue protrusion beyond the lips.

A 37-year-old male with known Down syndrome and epilepsy and taking carbamazepine was brought by his family to the emergency room with a large protruded tongue within 30 minutes of the incident (Figure 1).

**Figure 2:**
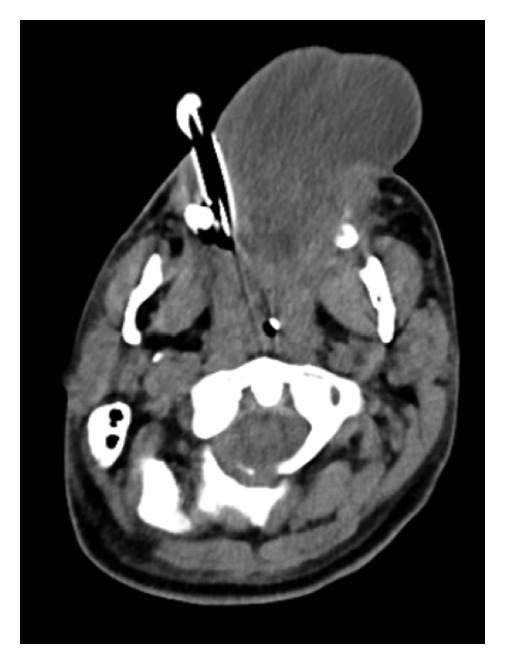
Axial cross section of a soft-tissue window computed tomography image with a tongue protrusion and endotracheal tube in place.

**Figure 3:**
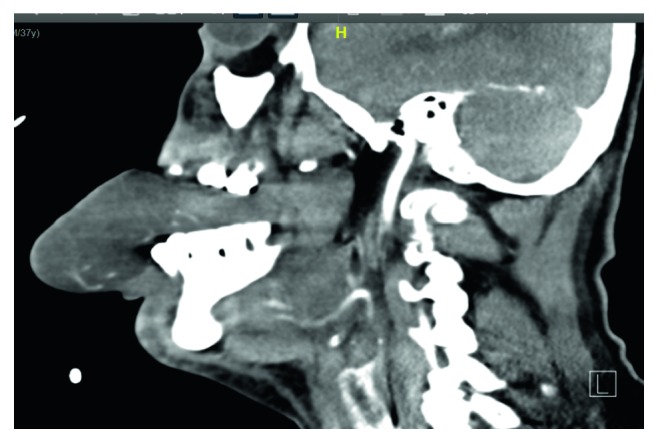
Sagittal cross section of a soft-tissue window computed tomography image with a tongue protrusion far beyond the lower lip.

The patient was vitally stable; however, he was slightly agitated. The otolaryngology team had been consulted immediately for further evaluation of the patient's status. History from the patient's family showed that they believed the patient was engaging in aggressive behavior, and he has a history of multiple tongue tractions leading to sudden severe tongue swelling. The examination was unremarkable, except for a huge protruded swollen tongue, which was diffused with no confined collection or sign of hematoma. Fixable nasolaryngoscopy relieved bilateral vocal cord movement, and there was no laryngeal edema. The neurology team was consulted to rule out active seizure, and they cleared him. The decision was to intubate the patient to secure the upper airway and then admit him to the intensive care unit (ICU). The patient consented to endotracheal intubation and possible tracheostomy in case intubation failed. The patient was successfully undergoing orotracheal intubation in the operating room. Then, a CT scan was done, which revealed some artifacts due to endotracheal intubation, with evidence of an enlarged diffused edematous hypertrophic tongue muscle protruded outside the oral cavity and deviated to the left side due to tube insertion from the right side (Figures 2 and 3). In addition, the CT scan showed no evidence of mass lesions, collection, abnormal enhancement in postcontrast series, or sizable lymphadenopathy. Thereafter, he was transferred while intubated to the ICU, and he was connected to mechanical ventilation. He was kept on midazolam, fentanyl, and dexamethasone at 8 mg intravenously for Q6h to relieve swelling, as well as pantoprazole for 4 days. Furthermore, a removable bite block was placed on the teeth to prevent tongue biting, and wet gauze was applied to the exposed part of the tongue. Subsequently, the swelling dramatically improved, and another nasolaryngoscopy was performed, which revealed no laryngeal or base of tongue edema. Formerly, he was extubated successfully and transferred to the ward. The patient was observed in the ward for 1 day and then discharged. The patient now has complete resolution of his symptom without any recurrence after 6 months of follow-up.

## 3. Discussion

Macroglossia is a condition that should always be taken seriously, as it may compromise the upper airway, particularly in traumatic cases due to the rapid onset of tongue and pharyngeal edema. In this situation, securing a definitive airway was the most important initial step. An upper airway obstruction is a life-threatening complication of traumatic macroglossia, particularly in severe cases and until the macroglossia is resolved. Therefore, the airway should be secured as the recovery period may be prolonged if it is not treated properly [11, 12].

Self-inflicted traumatic macroglossia is an extremely rare condition with no previous reported cases to our knowledge. However, few similar cases of traumatic macroglossia have been reported in the literature [7, 9, 10]. With regard to the management of macroglossia, it varies depending on the cause; in cases of congenital macroglossia and/or macroglossia secondary to a metabolic or vascular disease process, it is wise to treat the underlying disease in addition to conducting tongue reduction surgery. Meanwhile, in cases of traumatic macroglossia, a more conservative approach should be taken due to the tongue being an extremely compliant organ, while ensuring a definitively secured airway. For instance, Jakobson et al. reported the beneficence of using a bite raiser and muscle relaxant in reducing glossal edema secondary to seizure-induced traumatic macroglossia [7]. In addition, Lamond et al. reported a great result following the injection of a steroid into the base of the traumatic tongue swelling [13], as the mechanism of the steroid was able to stop the venous and lymphatic obstruction cycle and congestion, which may lead to further tongue edema [9]. In other cases of tongue entrapment in comatose patients, Theodoropoulos reported utilizing a small bite block, hand massages, and wet dressings to reduce traumatic macroglossia [14]. Meanwhile, another case of traumatic macroglossia secondary to a gunshot wound to the tongue was managed with maxillomandibular fixation for a period of 3 weeks until complete resolution of glossal edema [15]. There have also been reports of avoiding more trauma to the enlarged tongue using various prosthetic appliances, such as a modified bite guard [16]. In our review of the literature, we found that most authors report a recovery period of 1 week or greater to allow the swelling to subside [12]. Moreover, they reached agreement that the following three rules should be considered in acute settings to prevent further complications. First, airway management is important; therefore, securing the airway is a priority. Second, swelling should be controlled by administering steroids in all cases of gross tongue swelling, except hemorrhagic cases. Third, investigations should be done to determine the cause and treat it subsequently [17]. However, we managed our patient almost the same, with the only difference being that we avoided tracheostomy in contrast to other cases of traumatic macroglossia [7, 9, 10]. We were able to secure the airway successfully by rapid orotracheal intubation before further treatment was initiated. Yet, tracheostomy should always be considered to secure the airway, especially in the presence of the rapid deterioration of the clinical condition, depending on the persistence or continuity of the tongue swelling and protrusion.

To our knowledge, we believe this is the first reported case of self-induced traumatic macroglossia in an epileptic patient who was free from seizures when he presented with severe tongue swelling. This is the first report of the successful management of a patient with endotracheal intubation, corticosteroids, warm tongue compression, and the use of a bite raiser without the need for tracheostomy. We emphasize that such patients have difficult airways, and tracheostomy must be kept in mind. However, early detection and prompt management with rapid endotracheal intubation in the operating room as soon as possible using a bite block and corticosteroids to prevent more tongue injuries and swelling can enable a rapid recovery and prevent the patient from tracheostomy and its complications, as we have described.

## 4. Conclusion

Traumatic macroglossia is an extremely rare condition with the potential to compromise the upper airway and to threaten life; therefore, immediate airway management is crucial, followed by the use of steroids and a bite block to prevent further tongue injuries.
